# Supplementary material for: White Adipose Tissue Depots Respond to Chronic Beta-3 Adrenergic Receptor Activation in a Sexually Dimorphic and Depot Divergent Manner
Source: Cells. 2021 Dec 8;10(12):3453. doi: 10.3390/cells10123453 (PMC8700379; doi:10.3390/cells10123453)
Supplement: Supplementary file 1 [file cells-10-03453-s001.zip › cells-1349686-final-supplementary.pdf]

Table S1. Western blot antibodies.

WESTERN BLOT ANTIBODIES		
**Antibody**	**Source**	**Identifier**
Mouse Anti-Glut4	Cell Signaling Technology	#2213
Mouse Anti-OXPHOS Cocktail	Abcam	#110413
Rabbit Anti-PGC1alpha	Millipore Sigma	#516557
Rabbit Anti-UCP1	Millipore Sigma	#U6382
Rabbit Anti-GRP75	Cell Signaling Technology	#2816
Rabbit Anti-Adiponectin	Cell Signaling Technology	#2789
Rabbit Anti-Leptin	Abcam	ab117751
Rabbit Anti-Beta3 AR	Abcam	ab94506
Rabbit Anti-ATGL	Cell Signaling Technology	#2138
Rabbit Anti-HSL	Cell Signaling Technology	#4107
Rabbit Anti-FAS	Cell Signaling Technology	#3189
Rabbit Anti-Total AMPK	Cell Signaling Technology	#2532
Rabbit Anti-Phosphoserine AMPK	Cell Signaling Technology	#4185
Rabbit Anti-Phosphothreonine AMPK	Cell Signaling Technology	#2531
Rabbit Anit-ERalpha	Abcam	ab75635
Rabbit Anti-ERbeta	Abcam	ab3577
Rabbit Anti-Beta Actin	Abcam	ab8227
Goat Anti-Rabbit IgG	Cell Signaling Technology	#7074
Horse Anti-Mouse IgG	Cell Signaling Technology	#7076

Table S2. qRT-PCR primers.

qRT-PCR PRIMER SEQUENCES		
**Gene**	**Forward Primer (5` to 3`)**	**Reverse Primer (5` to 3`)**
Ppargc1a	GTTGCCTGCATGAGTGTGTG	TAGAGACGGCTCTTCTGCCT
Ucp1	CGCAGGGAAAGAAACAGCAC	GTTCCAGGATCCAAGTCGCA
Hspa9	TGATTGGAATTCCCCCAGCC	TGCACTGCCACGATTACTGT
Cidea	TGACCCCCCTCATACATCCA	GGCTACTTCGGTCATGGTTT
Prdm16	CAGCACGGTGAAGCCATTC	GCGTGCATCCGCTTGTG
Adipoq	TTCCATACCAGAGGGGCTCA	ACTCCGGTTTCACCGATGTC
Lep	AACCCTGTGCGGATTCTTGT	GGAGACTGACTGCGTGTGTG
Lipe	CCGAGATGTCACAGTCAATGGA	CAGGCCGCAGAAAAAAG
Fasn	GATTCAGGGAGTGGCTATTG	CATTCAGAATCGTGGCATAG
Esr1	TGCGAGCACATTCCTTCCTT	CTTCGTAGCATTTGCGGAGC
Esr2	GCATTGCCTGAACAAAGCCA	TTGGGACAGCACTCTTCGTC
Actb	AGACCTGTACGCCAACACAG	TTCTGCATCCTGTCGGCAAT

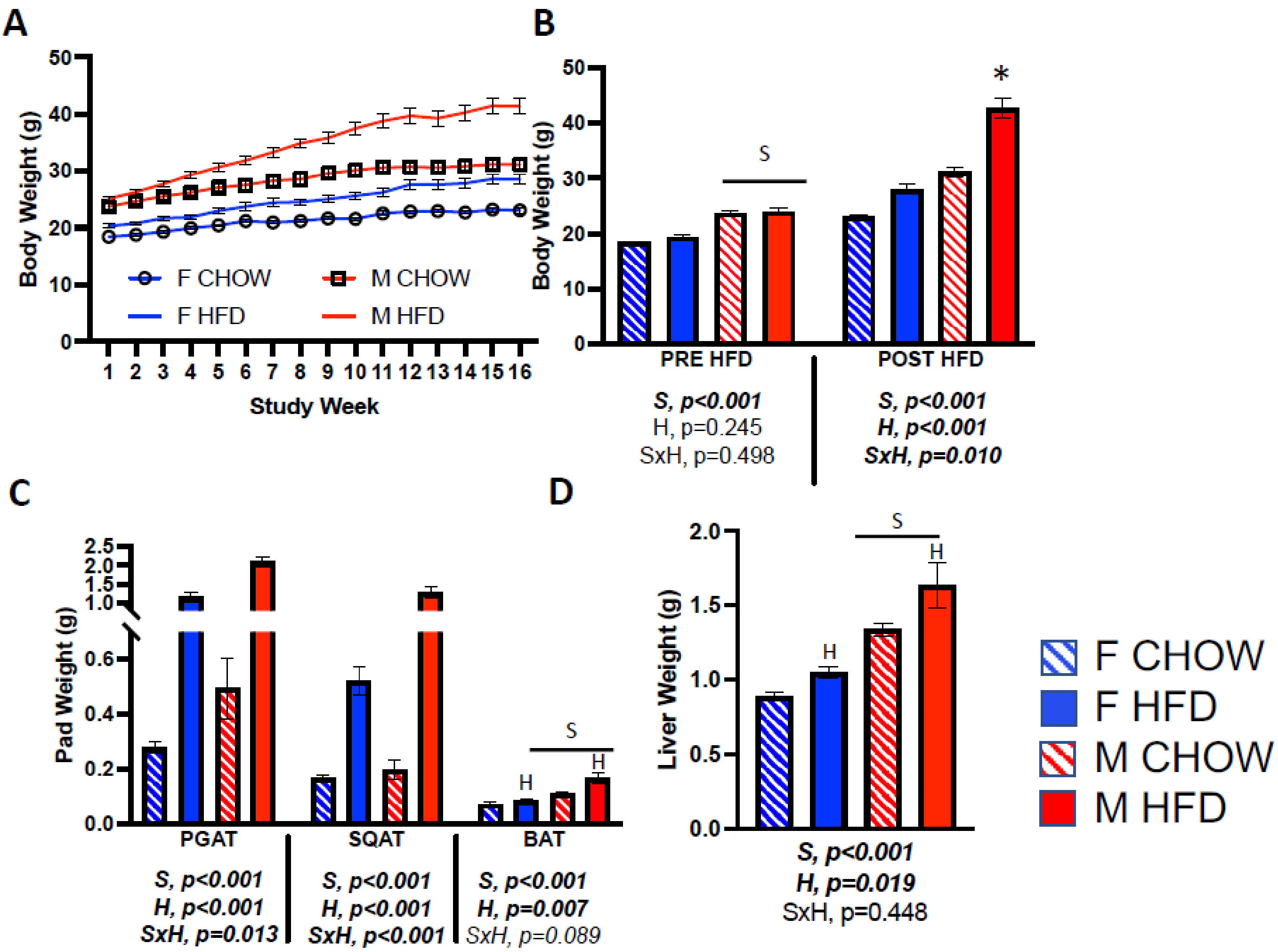

Figure S1. Sex differences in HFD-induced obesity. All mice are C57BL/6J, male (M) or female (F), fed standard chow (CHOW) or high fat diet (HFD). **(A)** Body weight throughout study; **(B)** Body weight pre- and post-HFD; **(C)** Pad weight for perigonadal (PGAT), subcutaneous (SQAT) and brown (BAT) adipose tissue depots; **(D)** liver weight. All data are presented as mean ± SEM. N = 7–15/group. Main effects of sex (S), high fat diet (H) and sex by diet interactions (S×H) were determined by 2×2 ANOVA and are presented below the respective figures. *p*-value < 0.05 was accepted as significant. * Significantly different from all other groups via post hoc Tukey's test.

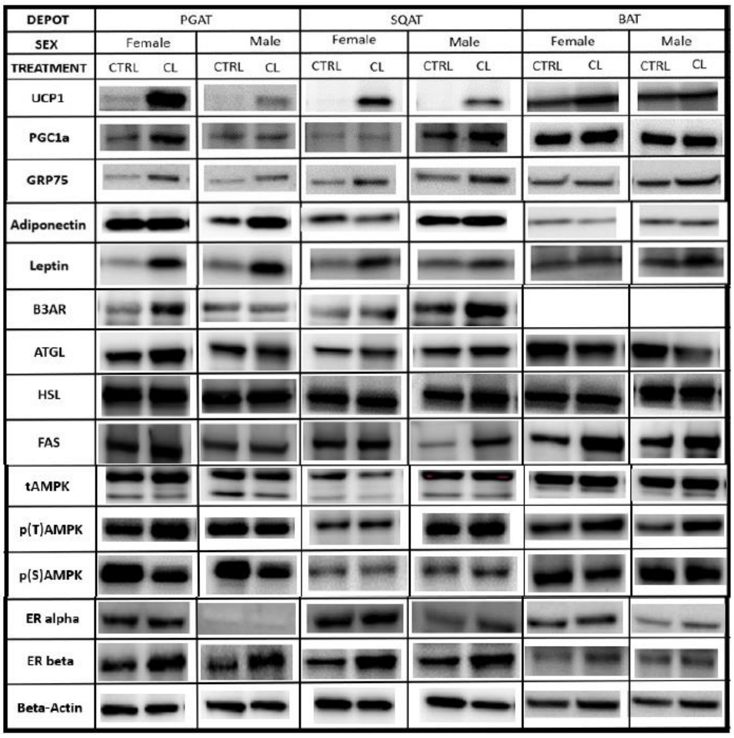

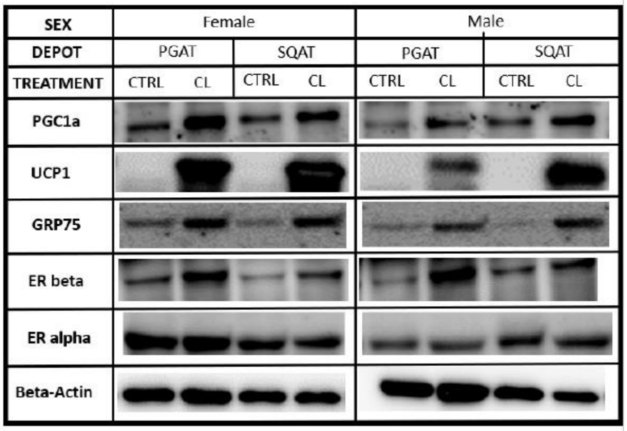

Figure S2. Representative Western blot images.
